# Enhanced Adjacency Matrix-Based Lightweight Graph Convolution Network for Action Recognition

**DOI:** 10.3390/s23146397

**Published:** 2023-07-14

**Authors:** Daqing Zhang, Hongmin Deng, Yong Zhi

**Affiliations:** School of Electronics and Information Engineering, Sichuan University, Chengdu 610064, China; daqingzhang@stu.scu.edu.cn (D.Z.); zhiyong@scu.edu.cn (Y.Z.)

**Keywords:** action recognition, skeleton data, CA-EAMGCN, feature selection, combinatorial attention

## Abstract

Graph convolutional networks (GCNs), which extend convolutional neural networks (CNNs) to non-Euclidean structures, have been utilized to promote skeleton-based human action recognition research and have made substantial progress in doing so. However, there are still some challenges in the construction of recognition models based on GCNs. In this paper, we propose an enhanced adjacency matrix-based graph convolutional network with a combinatorial attention mechanism (CA-EAMGCN) for skeleton-based action recognition. Firstly, an enhanced adjacency matrix is constructed to expand the model’s perceptive field of global node features. Secondly, a feature selection fusion module (FSFM) is designed to provide an optimal fusion ratio for multiple input features of the model. Finally, a combinatorial attention mechanism is devised. Specifically, our spatial-temporal (ST) attention module and limb attention module (LAM) are integrated into a multi-input branch and a mainstream network of the proposed model, respectively. Extensive experiments on three large-scale datasets, namely the NTU RGB+D 60, NTU RGB+D 120 and UAV-Human datasets, show that the proposed model takes into account both requirements of light weight and recognition accuracy. This demonstrates the effectiveness of our method.

## 1. Introduction

In recent years, the rapid development of deep learning has resulted in its great success in computer vision tasks [1]. As a research hotspot in this field, action recognition has been widely used in human–computer intelligent interaction, virtual reality, video surveillance and other practical applications [2,3,4]. human action recognition extracts discriminative behavior features to fully describe the temporal dynamics and spatial structure of human motion [5]. Currently, however, due to the high complexity and variability of human action, recognition models cannot fully meet the practical requirements of both light weight and recognition accuracy.

At present, skeleton-based human data representation has been extensively studied and applied in human action recognition. Firstly, skeleton data, as a natural representation of human joint positions in the form of 2D or 3D coordinates, can be better extracted from depth sensors like Microsoft Kinects [6]. Secondly, human skeleton data contain relatively richer behavior characteristics with a compact data form [7] for describing human motion. In addition, skeleton-based data representations are less susceptible to lighting, camera angle and other background changes, showing more robustness. The many advantages of skeleton-based human data representation have promoted the exploration and utilization of the information features in skeleton motion sequences for action recognition.

Traditional methods used to deal with skeleton data mainly include convolutional neural networks (CNNs) [8,9] and recursive neural networks (RNNs) [10,11]; current mainstream action recognition methods utilize graph convolutional networks (GCNs) [12,13]. CNN-based methods model the skeleton data into pseudo-images using manually designed transformation rules. For example, Kim and Reiter [9] linked joint coordinates and used a 1D residual CNN to identify skeleton sequences, providing a method to learn and interpret 3D human action recognition. RNN-based methods usually model the skeleton data in spatial and temporal dimensions as a coordinate vector sequence. For example, Du et al. [10] constructed a hierarchical bidirectional RNN model to capture the sequence features between different body parts. However, human skeleton data are naturally connected in the form of graphs, rather than in the form of 2D grids or vector sequences. Therefore, it is difficult for CNN-based and RNN-based methods to represent the topological structure of skeleton data and fully express the spatial structure information between human joints. GCN is a universal deep learning framework, which can be directly applied to non-Euclidean structured data, in comparison with other mature models [14]. More recently, Yan et al. [12] first constructed a spatial–temporal graph convolution network (ST-GCN) for skeleton-based human action recognition. They innovated the research methods of human action recognition by modeling the human skeleton data as a spatial–temporal graph.

Inspired by ST-GCNs, more and more researchers have applied GCNs to skeleton-based action recognition tasks. Si et al. [15] proposed an AGC-LSTM model, wherein the graph convolution module is embedded into a multi-layer LSTM module which is able to improve the graph convolution network’s performance of extracting the features of joints in the temporal dimension. Shi et al. [16] constructed a two-stream action recognition model, improving the limitations of input data with only node features, which can be utilized to adaptively learn the topological structure in the spatial–temporal dimension and enhance the recognition ability of the model. Compared with previous studies, PA-ResGCN-N51 [17] and EfficientGCN-B0 [18] innovatively used three early fused input features to reduce the complexity and number of parameters and introduced a residual network to increase the stability of the model. Xie et al. [19] proposed a new strategy for constructing graph convolution kernels that mainly automatically adjusted the number of graph convolution kernels according to the complexity of the topology. Wang et al. [20] calculated the weight values of the edges between non-physically connected nodes in a human skeleton graph according to the node distance, increasing the sensitivity field of the GCN. At the same time, a modified partitioning strategy is adopted to extract a large amount of non-adjacent joint information from the model.

These models based on GCNs can significantly improve recognition accuracy, but they also have some limitations: (1) The adjacency matrix set according to predefined human body topology is usually fixed in all graph convolutional layers and input samples, which is not the best choice for action recognition task. Meanwhile, graph convolution kernels artificially defined based on the adjacency matrix can only extract the feature information of neighbor nodes, and it is difficult to capture the feature information between two nodes that are far apart in the topology of the human body graph, which lacks the ability to perceive the features of nodes globally. (2) The multi-stream frame model [16], by making use of first-order information (joint coordinates) and second-order information (motion speed and bone characteristics) of skeleton data, has achieved good performance. However, the behavior of the human body is a consecutive motion stream in multiple frames, in which each sub-stage has different characteristic information. The direct fusion of first-order information and second-order information of skeleton data ignores the fact that different input features have different degrees of importance to different action samples, leading to the redundancy of feature information and introducing confusion regarding the identification features extracted by the model. (3) The effectiveness and necessity of attention mechanisms in action recognition tasks have been demonstrated. Some related works [17,21] used single attention mechanism with GCNs so as to discover the relationship between joints and between frames and identify the differences between action samples. In a GCN model of a hierarchical architecture like CNN, different graph convolutional layers contain different semantic information; single attention mechanisms lack the ability to understand the multi-layer semantic information of all graph convolutional layers, smoothing the output features of the model.

To address the above issues, we propose an enhanced adjacency matrix-based graph convolutional network with a combinatorial attention mechanism (CA-EAMGCN) to improve the extraction of skeleton data features. Specifically, our main contributions in proposing the CA-EAMGCN model are as follows: (1) A novel method to construct an adjacency matrix is proposed. The new adjacency matrix is optimized for different graph convolution layers, making up for the shortcoming that graph convolution kernels can only extract the features of neighbor nodes, enhancing the ability of the model to capture the features of global nodes and provide global regularization for graph learning. (2) A feature selection fusion module (FSFM) is designed, which can adaptively calibrate the fusion ratio of multiple input features to enhance the differences of input data and reduce the amount of redundant feature information. (3) A combinatorial attention mechanism is proposed to enhance the model’s ability to understand the semantic information contained in different graph convolution layers. Specifically, in the multi-input branch stage of the model, a spatial–temporal (ST) attention module is designed to process the semantic information at the spatial and temporal levels of different graph convolution layers; in the mainstream network stage of the model, a limb attention module (LAM) is designed to process the joint semantic information between local joints in the human body topology in one graph convolution layer.

In order to verify the effectiveness of our proposed model, we conduct extensive experiments on three large-scale datasets, NTU RGB+D 60 [22], NTU RGB+D 120 [23] and UAV-Human [24]. The experimental results show that our model achieves good performance for all datasets. The proposed model is lightweight while ensuring recognition accuracy.

The main contributions of this paper are outlined as:

- A novel method to construct an enhanced adjacency matrix is proposed, improving the ability of capturing the global features among joints and providing global regularization for graph learning;
- A feature selection fusion module is designed to provide a more suitable fusion ratio for multi-stream input features;
- A combinatorial attention mechanism is proposed to enhance the model’s ability to understand the semantic information in different graph convolution layers;
- Extensive experiments on three large-scale datasets, namely NTU RGB+D 60, NTU RGB+D 120 and UAV-Human, verify the validity of our proposed method.

## 2. Related Works

### 2.1. Skeleton-Based Action Recognition

In recent years, skeleton-based action recognition methods with more compact data representation have attracted more and more attention. In traditional methods of skeleton-based action recognition, classification was usually performed according to the movement trajectory of human joints after extracting hand-crafted behavior features [25,26,27]. For example, Vemulapalli et al. [26] used rotation and shift transformation to encode a skeleton in a location group; Fernando et al. [27] used the rank pooling method to represent data via the parameters of the ranking function. The ability of hand-designed features for action recognition was limited because they did not make good use of the spatial relations of human joints. Moreover, the recognition models constructed based on hand-designed behavioral features were set for specific applications and were difficult to extend to other applications and had poor flexibility.

With the rapid development of deep learning and its great success in computer vision tasks, researchers have applied deep learning methods to skeleton-based action recognition and achieved good results. Skeleton-based action recognition models using deep learning methods can be divided into three categories, namely CNN-based methods, RNN-based methods and GCN-based methods.

The first category involves models based on CNNs. For example, Liu et al. [28] proposed a new transformation rule for skeleton data modeling, designed ten kinds of spatial–temporal images and used visual and motion enhancement methods for data argumentation before training. Li et al. [29] used data enhancement strategies and multi-scale residual networks to achieve skeleton-based action recognition.

The second category involves models based on RNNs. As a modification of RNN networks, LSTM networks were later used for skeleton-based action recognition because of its better performance in longer sequences. For example, Song et al. [30] embedded a spatial-temporal attention module in a LSTM-based model to improve the weights of important joints in a skeleton sequence in order to enable the model to automatically identify the skeleton sequence in spatial and temporal dimensions. Zhang et al. [31] introduced a view conversion mechanism to a model based on a LSTM network to help the model automatically transform skeleton data into angles which were conducive to action recognition.

The two above-mentioned kinds of models usually represent skeleton data in a grid-like manner or a vector sequence, which often leads to insufficient extraction of spatial structure information of skeleton data. However, the third category of model based on GCNs can naturally process skeleton graphs of the human body and better extract spatial structure information. Yan et al. [12] first proposed a spatial–temporal graph convolutional network (ST-GCN) to apply GCNs to skeleton-based action recognition. Following this, GCN-based methods became more and more used in the research of skeleton-based action recognition. On the basis of ST-GCNs, some researchers have made many improvements. Li et al. [32] integrated posture prediction into the task of action recognition to help their model capture more detailed action features through self-supervision. Song et al. [33] extended GCNs by learning discriminative features from joints less activated previously. Nan et al. [34] combined a GCN and a temporal convolutional network (TCN) to design an efficient pipeline for solving the problem of human action recognition using skeletal data. 

### 2.2. Multi-Stream Framework Model

In addition to feature vectors attached to each vertex containing the 2D or 3D coordinates of a joint, the speed of joints and the length and direction of bones between pairs of joints are also characteristic information. Simonyan and Zisserman [35] applied optical stream field information to RGB-based action recognition to construct a two-stream convolution network and proved its usefulness. Inspired by this, Shi et al. [21] used joint coordinates to define the length and direction of bones and took the coordinate differences between joints and bones in two continuous frames as motion information to build a GCN-based multi-stream network. In order to reduce the complexity of the model, Song et al. [17] fused the preprocessed multi-stream input data in the early stage and introduced a residual network to increase the stability of the model.

### 2.3. Attention Mechanism

Attention mechanisms have become an important part in computer vision tasks, so it is necessary to introduce them into skeleton-based action recognition. In these tasks, a certain action usually consists of multiple continuous motion segments, each of which is usually associated with a key subset of joints, so different substages have different levels of importance for recognition. This critical information can be utilized through an attention mechanism to improve the recognition performance of models. Baradel et al. [36] established a spatial–temporal attention mechanism using human posture information and introduced it into an RGB-based action recognition network. Song et al. [30] introduced an attention mechanism into skeleton-based action recognition for the first time; the established spatial–temporal attention module could distinguish between joints in each frame and generate the corresponding attention matrix. Shi et al. [21] introduced a spatial–temporal-channel attention module for each graph convolution layer, allowing their model to provide different spatial, temporal and feature perspectives so as to adaptively calibrate the activation of joints, frames and channels in different data samples. Song et al. [17] utilized a part-wise attention module to find the most important joints in an action sequence and calculate the attention weights of different joints in the sequence to further improve the distinguishing ability of features.

## 3. Methodology

In this section, we illustrate the main steps, framework and key modules of our proposed method.

### 3.1. Graph Structure

By definition, the typical graph structure of an undirected graph can be represented by *G* = (*V*, *A*, *X*), where *V* = {*v*_1_, *v*_2_, ···, *v_N_*} represents a set of nodes, and *A* ∈ {0, 1}*^N×N^* is an adjacency matrix. If there is a connection between *v_i_* and *v_j_*, then *A_ij_* = 1, otherwise *A_ij_* = 0. *X* ∈ R*^N×D^* represents the feature subset of each node, where *N* is the number of nodes and *D* is the number of feature channels.

### 3.2. Data Preprocessing

According to the previous research methods [16,17], data preprocessing is a very important phase for skeleton-based action recognition. In order to make the preprocessed skeleton data more consistent with human movement, inspired by Song et al. [17], we used a new preprocessing method to divide the skeleton data into three factors: (1) joint position, (2) motion speed, (3) bone characteristics. We assumed that the original 3D coordinate set of a skeleton sequence is $X=\{x\in R^{C\times T\times N}\}$, where *C*, *T* and *N* represent the original 3D coordinates of nodes, frames and joints, respectively. The relative positions $P=\{p_{i}\left|i=1,2,\cdots ,N\right.\}$ can be obtained, where

(1) $$p_{i}=x[:,:,i]-x[:,:,c]$$

(2) $$x[:,:,c]=\sum_{i=1}^{V}\frac{x[:,:,i]}{V}$$

The original 3D coordinate set and relative position set of nodes are connected into a single sequence as the input branch of joint positions. Then, two sets of velocities can be obtained using the original coordinate set of the skeleton sequence $F=\{f_{t}\left|t=1,2,\cdots ,T\right.\}$ and $S=\{s_{t}\left|t=1,2,\cdots ,T\right.\}$ where

(3) $$f_{t}=x[:,t+2,:]-x[:,t,:]$$

(4) $$s_{t}=x[:,t+1,:]-x[:,t,:]$$

A feature vector obtained by connecting the two groups of velocity *F* and *S* is used as the input branch of the velocity. Finally, the bone feature input branch includes bone length $\Gamma =\{l_{i}\left|i=1,2,\cdots ,N\right.\}$ and the angle of bone $\Psi =\{\varphi_{i}\left|i=1,2,\cdots ,N\right.\}$ where

(5) $$l_{i}=x[:,:,i]-x[:,:,i_{adj}]$$

(6) $$\varphi_{i,w}=\arccos(\frac{l_{i,w}}{\sqrt{l_{i,x}^{2}+l_{i,y}^{2}+l_{i,z}^{2}}})$$

*i_adj_* refers to the adjacent joint of the *i*-th joint, and *w* is the 3D coordinate of the central node of the human skeleton.

### 3.3. Spatial–Temporal Graph Convolutional Network (ST-GCN)

**Figure 1:**
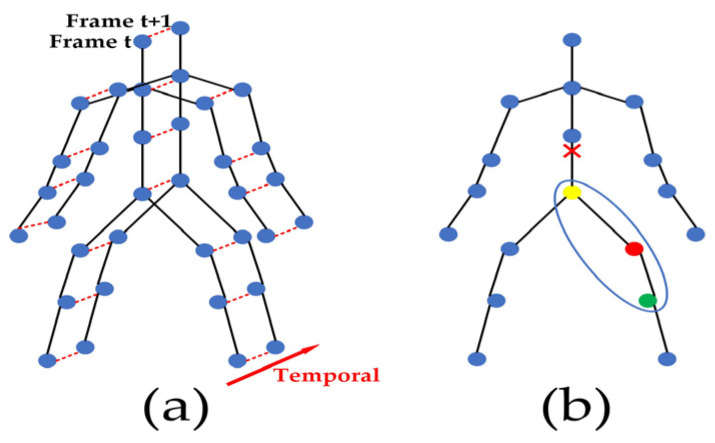
The skeleton graph of human body. (**a**) Spatial-temporal skeleton graph. (**b**) Neighbor node partitioning strategy (different colors represent different subsets).

Yan et al. [12] proposed a human spatial–temporal skeleton graph used to simulate structured information between joints in spatial and temporal dimensions which referred to the joints in the same frame and the same joints in all frames, respectively. Figure 1a is an example of a constructed spatial–temporal skeleton graph where joints are represented by vertices and their natural connections in the human body are represented by spatial edges, i.e., the black lines in Figure 1a. Each temporal edge is the connection of corresponding joints in two consecutive frames, i.e., each red dotted line in Figure 1a. The ST-GCN was composed of 9 basic blocks, each of which performed spatial and temporal dimensional convolution. The spatial dimension convolution operation of each frame in the skeleton sequence data could be expressed as:

(7) $$f_{out}=\sum_{k=1}^{K_{v}}W_{k}f_{in}(\Lambda_{k}^{-\frac{1}{2}}{\bar{A}}_{k}\Lambda_{k}^{-\frac{1}{2}})⊗M_{k}$$

where K*_v_* represents the size of the convolution kernel in spatial dimensional convolution, namely the number of matrices. In the ST-GCN, the neighbor nodes of each node were divided into three categories according to the respective partition strategy. Figure 1b shows the partition strategy of neighbor nodes, where the red × sign represents the center of gravity of the human skeleton. Specifically, each node and its neighbors form a neighbor set (the region circled by the blue line in Figure 1b). The partition strategy divided the neighbor set into three subsets: (1) the root node itself (the red circle); (2) a centripetal subset containing adjacent nodes that are closer to the center of gravity of the skeleton than the root node (yellow circle); and (3) a centrifugal subset containing adjacent nodes further away from the center of gravity of the skeleton than the root node (green circle). Therefore, there were three types of adjacency matrices of graphs, K*_v_* = 3 in Formula (7). f*_in_* and f*_out_* denote input and output features and ⊗ denotes element multiplication. ${\bar{A}}_{k}=A_{k}+I$, where ${\bar{A}}_{k}$ represents the K-interface adjacency matrix; it is used to extract the connection vertices in a specific subset from the input feature f*_in_* as the corresponding weight vector. A*_k_* represents the adjacency matrix without self-connection, I represents the identity matrix and $\Lambda_{k}^{-\frac{1}{2}}$ is used for the normalization of ${\bar{A}}_{k}$. W*_k_* and M*_k_* are learnable parameters, W*_k_* is the $C_{out}\times C_{in}\times 1\times 1$ weight vector for the 1×1 convolution operation and M*_k_* can be used to adjust the importance level of each edge.

For temporal dimension convolution, the number of neighbors of each node was 2, so traditional convolution operations could be used to achieve feature extraction in the temporal dimension. Specifically, a convolution layer of L×1 was designed to extract contextual features between adjacent frames, where *L* is a predefined parameter used to define the length of the time window. By performing convolution operations in spatial and temporal dimensions, a basic graph convolution module was constructed.

### 3.4. The Proposed Model—Enhanced Adjacency Matrix-Based Graph Convolution Network

Although the ST-GCN model has become the baseline network, it has certain limitations: (1) The partitioning strategy is not the best choice for action recognition tasks. As shown in Figure 1b, there are few neighbor nodes surrounding root nodes, which means that there are many 0 values in the adjacency matrix A*_k_* based on the partitioning strategy. (2) The dot multiplication operation between the input feature f*_in_* and the adjacency matrix A*_k_* means that if one element in the adjacency matrix A*_k_* is 0, the result of dot multiplication between them will always be 0 regardless of the value of the input feature f*_in_*; as a result, the graph convolutional network can only aggregate the feature information of neighbor nodes of the root node, which leads to a lack of awareness of the global node information of the model. A similar situation occurs for the mask matrix M*_k_*.

In order to address this issue, we propose a parameterized quasi-adjacency matrix, mainly aimed at assigning different virtual connection strengths to nonadjacent nodes in the skeleton graph in the corresponding positions (with 0 s) of the original adjacency matrix. We then combined this parameterized quasi-adjacency matrix with the original adjacency matrix to form a new adjacency matrix. The values of the elements in the parameterized quasi-adjacency matrix were not obtained by specific calculation but given certain initial values before the model started training, which can be adjusted and optimized according to the training situation to cope with different action categories; the values of the elements in the training process were restricted between 0 and 1. This not only maintains the predefined skeleton graph topology but also establishes distant dependency relationships in the predefined graph topology and extends the feature extraction capability of the graph convolutional network from adjacent nodes to all nodes, so as to realize the enhanced adjacency matrix. 

Specifically, the construction of the quasi-adjacency matrix and the implementation of the adjacency matrix enhancement strategy are as follows: Firstly, the adjacency matrix ${\bar{A}}_{k}=A_{k}+I$ is obtained according to the predefined human skeleton graph, where A*_k_* represents the adjacency matrix without self-joining and I represents the identity matrix, *k* = 1, 2, 3, and the quasi-adjacency matrix QAM(${\bar{A}}_{k}$) with the same dimension as ${\bar{A}}_{k}$ is also created at the same time. Then, the parameterized quasi-adjacency matrix E*_k_* is added to the adjacency matrix ${\bar{A}}_{k}$. The above steps can be expressed by the following formulas:

(8) $$E_{k}=\mathrm{Para}(\mathrm{QAM}({\bar{A}}_{k})),k=1,2,3$$

(9) $$G_{k}={\bar{A}}_{k}+E_{k},k=1,2,3$$

Finally, we achieve the enhanced adjacency matrix G*_k_*. Therefore, the graph convolution formula based on the adjacency matrix enhancement is modified as Formula (10):

(10) $$f_{out}=\sum_{k=1}^{K_{v}}W_{k}f_{in}(\Lambda_{k}^{-\frac{1}{2}}G_{k}\Lambda_{k}^{-\frac{1}{2}})⊗M_{k}$$

**Figure 2:**
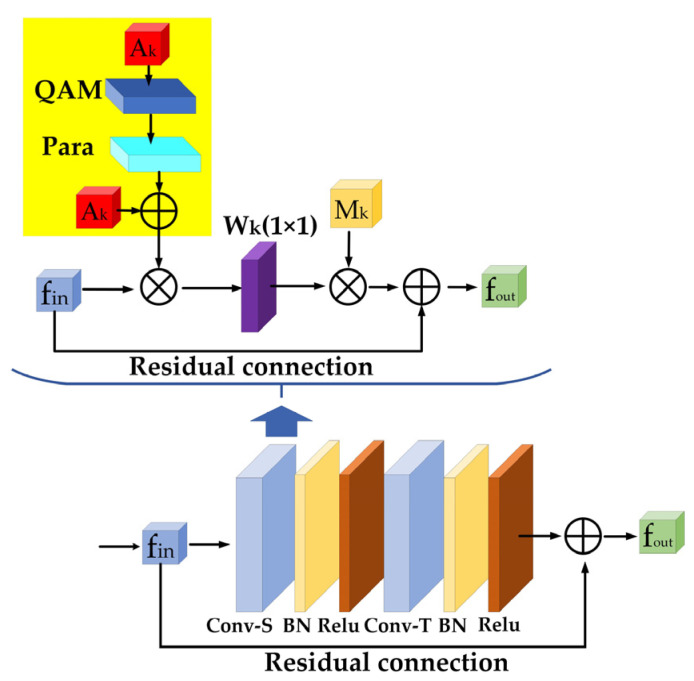
The enhanced adjacency matrix-based graph convolution network (EAMGCN). The yellow square area in the figure shows the building process of the enhanced adjacency matrix G*_k_*.

Figure 2 shows the architecture of the EAMGCN unit. The value of elements in the new adjacency matrix G*_k_* represent whether there is a connection between joints, as well as the strength of the connection. At the same time, as a self-learning method of connection strength between nodes, the adjacency matrix enhancement strategy can effectively reduce both the calculation amount and the number of parameters of the model. When using the adjacency matrix enhancement strategy, we need to pay attention to initializing the elements in the created quasi-adjacency matrix E*_k_* as 0, so that the adjacency matrix A*_k_* dominates the initial stage of training, which is conducive to stabilizing the training process and improving the stability of the model. In conclusion, the enhanced adjacency matrix strategy not only maintains the predefined graph topology but also makes up for the shortcoming that there is no new connection in the predefined graph topology, enhances the model’s ability to capture the features of global nodes, and reduces the complexity of the model. In addition, the bottleneck block structure which has shown its effectiveness in the work of Song et al. [17] was introduced into the EAMGCN to further improve the efficiency of the model.

### 3.5. Feature Selection Fusion Module

So far, multi-stream framework models have been used with great potential to extract rich behavior features and achieve excellent performance in tasks of action recognition. For example, Shi et al. [16] used joint information to model a two-stream frame model, which significantly improved accuracy of recognition. The essence of multi-stream frameworks is data enhancement, which provides rich feature information for models. However, the richer the feature information, the greater the number of parameters to be adjusted during training, which increases calculation cost and the difficulty of parameter optimization. An early fused multi-input branch structure was proposed [17], where the fused features were input into a mainstream network, significantly reducing the complexity of the model while retaining rich input features. However, human behavior contains a series of continuous motions, and an action stream contains movement information in multiple stages; different input characteristics have different degrees of importance for different sub-stages of movement. The above-mentioned early fused multi-input branch architecture could reduce the complexity of the model and the number of training parameters, but its direct fusion of multiple input features still caused input feature redundancy, which indirectly led to difficulty in extracting rich identification features, thus affecting the recognition accuracy. Sometimes, the motion speed of adjacent nodes is more crucial for distinguishments like that between “walk” and “run”, so in this situation, the model should pay more attention to the input feature of the motion speed of nodes during training rather than other more redundant information.

To solve the above issue, we designed a module called FSFM, its essence is a channel attention. Via the FSFM, the model can match an appropriate fusion ratio for each input feature before multi-input feature fusion, enriching the discriminant features of the recognition model. The calculation formula can be expressed as in (11):

(11) $$f_{out}=\alpha (\mathrm{AvgPool}(f_{in})W)$$

where f*_in_* and f*_out_* represent input and output features, AvgPool() represents the average pooling operation, *α* represents the sigmoid function and W represents the weight vector of the convolution operation.

### 3.6. Combinatorial Attention Mechanism

From the perspective of data structure, skeleton data comprise a temporal sequence composed of 3D coordinates of human joints. For different action categories, different attention mechanisms should be adopted based on the joints, frames and channels of skeleton sequences in different training conditions in order to extract the relatively critical features. For instance, the spatial–temporal-channel attention module was introduced to determine the distinguishing features in skeleton sequences based on data structures [21]. On the other hand, movement has integrity and continuity. When a person is moving, movement is usually dominated by some parts of his body with other body parts’ cooperation to complete a series of actions. For example, when it comes to drinking water, the upper limbs of the human body are definitely more important than the lower limbs. Therefore, we need to pay different attention to the node features of different limb parts in skeleton sequences during human movement. Some skeleton-based action recognition methods usually use a single attention mechanism to discover the key information of skeleton sequences and do not comprehensively consider the node features of human movement from multiple perspectives. Moreover, different graph convolution layers contain different semantic information; if only a single attention mechanism were taken into account, the model would become dependent on the single attention mechanism and smooth the output features with the deepening of the graph convolution layers.

In view of above-mentioned facts, we propose a combinatorial attention mechanism that focuses on the node features of human motion from multiple perspectives and enhances the understanding ability of the model regarding the semantic information contained in different graph convolution layers. Specifically, in the multi-input branch stage of the model, a spatial–temporal attention module is designed to determine the key node features in skeleton sequences from the perspective of data structure and process the semantic information in the spatial and temporal dimensions of the graph convolution, so that rich feature information can be extracted prior to the stage of feature fusion. The calculation formula can be expressed as:

(12) $$f_{out}=\beta (\beta (\mathrm{AvgPool}(f_{in})W_{s})W_{t})$$

where f*_in_* and f*_out_* represent input and output features, AvgPool() represents the average pooling operation, *β* represents the sigmoid function and W*_s_* and W*_t_* represent the weight vectors of convolution operations in the spatial and temporal dimensions, respectively.

**Figure 3:**
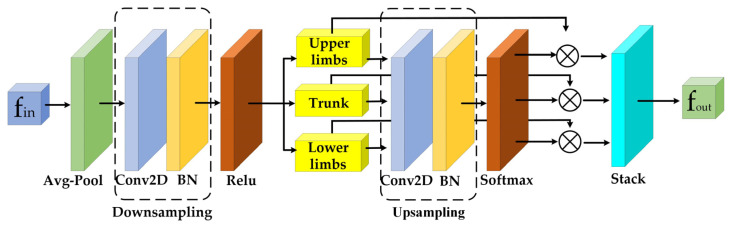
The limb attention module.

In addition, in order to determine those body parts carrying more effective information in skeleton sequences of human movement, a limb attention module is designed in the mainstream network stage of the model, and its structure is shown in Figure 3. In this new design, relations between local and global semantic information in graph convolutional layers can be better learned. The implementation steps are as follows: (1) All node features of the human body are averagely pooled in the temporal dimension, and then node features are extracted in the spatial dimension through the 2D convolution layer plus the BatchNorm layer. (2) The skeleton is divided into trunk, upper limb and lower limb sections according to the human body structure. The trunk includes the head node and spine node of the skeleton graph, the upper limb section includes the left and right arm nodes, and the lower limb section includes the left and right leg nodes. This allows for a more compact representation of the skeleton graph of the human body and a better understanding of human behavior by classifying the symmetrical body parts into the same part corresponding to the coordination features of human movement. (3) The attention matrix is calculated by the 2D convolutional layer plus a BatchNorm layer, and the body parts are determined by the Softmax classification function. (4) the node features of the three sections are connected into an output feature matrix with different attention weights. The limb attention module can be represented by the following formulas:

(13) $$f_{l}=f_{in}(l)⊗\lambda (\mu (\mathrm{Pool}(f_{in})W)W_{l})$$

(14) $$f_{out}=stack(\{f_{l}\left|l=1,2,3\right.\})$$

where f*_in_* and f*_out_* represent the input and output features, ⊗ represents element-level multiplication, Pool() represents the adaptive average pooling operation, *λ* represents the Softmax function, *μ* represents the ReLU activation function, W denotes the weight vector of convolution operations on all node features and W*_l_* represents the weight vector of convolution operations on each limb part.

In summary, the input branch first obtains rich features through the spatial–temporal attention module and then fuses them in a proper ratio in the feature selection fusion module proposed in Section 3.5. The fused features are further extracted from the mainstream network via the limb attention module. Finally, through the combination of these two attention modules, we obtain the proposed enhanced adjacency matrix-based graph convolutional network with a combinatorial attention mechanism (CA-EAMGCN) which significantly improves recognition performance.

### 3.7. Model Architecture

**Figure 4:**
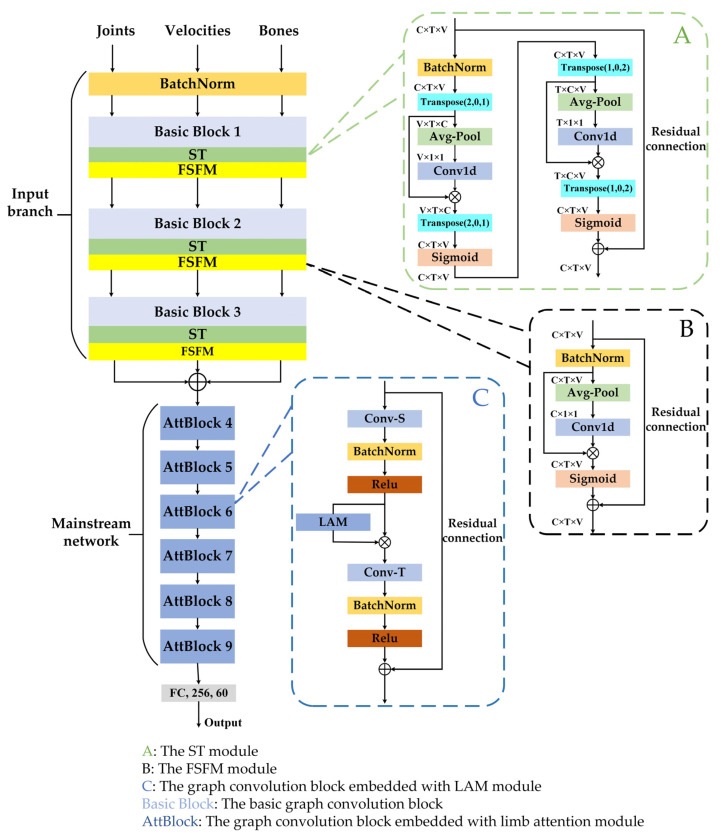
Model architecture.

Figure 4 shows the overall structure of the network. It is mainly composed of a multi-input branch module and a mainstream network. The multi-input branch module comprises 3 stacked basic blocks, and the numbers of output channels in each block are 64, 64 and 32, respectively. The mainstream network consists of 6 stacked basic blocks with 128, 128, 128, 256, 256 256 channels, respectively. Figure 2 shows the structural composition of each basic block, with the LAM embedded only in the basic block of the mainstream network. We start by adding a BatchNorm layer to normalize the input data, and end with a fully connected layer to classify action categories. The area bordered by the green dashed line indicates the spatial–temporal attention module, the area bordered by the black dashed line represents the feature selection fusion module and the area bordered by the blue dashed line represents the graph convolution basic block embedded with the limb attention module.

## 4. Experiments

In this section, we evaluate the performance of the proposed CA-EAMGCN model on three large datasets NTU RGB+D 60 [22], NTU RGB+D 120 [23] and UAV-Human [24]. To achieve these results, we conducted a large number of ablation experiments and extensive comparison experiments with the proposed model and other skeleton-based action recognition methods.

### 4.1. Datasets

#### 4.1.1. NTU RGB+D 60 Dataset

NTU RGB+D 60 is a common indoor action recognition dataset. The actions in the dataset contain 60 types of movements, and a total of 56,880 videos of human movements were taken as data samples. Forty different volunteers were recruited to perform the movements while each video was filmed by three cameras at the same height with different horizontal views. A Kinect v2 depth sensor was used to obtain the 3D coordinates of 25 joints that constructed the human skeleton, forming a skeleton sequence with no more than two human skeletons in each frame. The authors of this dataset recommended two different classification criteria: (1) cross-subject (CS): the training set contains 40,320 video samples from 20 volunteers, and the test set contains 16,540 video samples from the rest of the volunteers; and (2) cross-view (CV): the training set contains 37,920 video samples from two camera views, and the test set contains 18,960 video samples from another camera view.

#### 4.1.2. NTU RGB+D 120 Dataset

This dataset is the largest indoor motion recognition dataset at present. It is an extended version of the NTU RGB+D 60 dataset. This dataset was obtained from 106 volunteers, including 120 kinds of actions, taking a total of 114,480 videos of human movements as data samples. Thirty-two kinds of collection IDs were set for the dataset, and the locations and backgrounds changed with different collection ID settings. Similarly, the authors recommended two different classification criteria: (1) cross-subject (Csub120): the training set contains 63,026 video samples from 53 volunteers, and the test set contains 50,922 video samples from the rest of the volunteers; and (2) cross-setup (Cset120): The training set contains 54,471 video samples whose IDs were set to even numbers. The test set contains 59,477 video samples with odd IDs.

#### 4.1.3. UAV-Human Dataset

This dataset was collected by a flying drone in multiple urban and rural areas during the day and night over 3 months. The UAV-Human dataset contains 67,428 multi-mode video sequences and 119 targets for action recognition, 22,476 frames for posture classification, 41,290 frames and 1144 frames for gender reidentification and 22,263 frames for attribute identification. it covers a wide variety of subjects, backgrounds, lighting, weather, occlusion, camera movements and drone flying attitudes. It is a comprehensive and challenging benchmark able to facilitate UAV-based research into human behavior understanding, including motion recognition, posture estimation, re-recognition and attribute recognition.

### 4.2. Experimental Settings

All experiments were conducted in the PyTorch deep learning framework. Regarding the basis of the node partitioning strategy proposed for ST-GCNs, we adopted the spatial configuration partitioning strategy, so the maximum map distance K*_v_* mentioned in Section 3.3 was set to 3, and the temporal window length *L* was set to 9. Each skeleton sequence was set to 300 frames. For samples less than 300 frames, we repeated the sample data until it reached 300 frames. The stochastic gradient descent (SGD) method with a Nesterov momentum of 0.9 and weight attenuation of 0.0002 was used to optimize and adjust the parameters, and the cross-entropy was selected as the loss function. A total of 50 training epochs were carried out. The warm-up training strategy was adopted in the first five training epochs, and the initial learning rate was set at 0.1, which decayed by 10 from the 40th iteration. Our model was trained on a GTX 3060 GPU.

### 4.3. Ablation Study

In this section, we describe tests of the validity of each module in our CA-EAMGCN model based on the NTU RGB+D 60 dataset.

#### 4.3.1. Enhanced Adjacency Matrix Strategy

In this subsection, we describe our testing of the effectiveness of the enhanced adjacency matrix G*_k_* described in Section 3.4, and the experimental results are shown in Table 1. We tested the contribution of the enhanced adjacency matrix G*_k_* to the original ST-GCN using three input features (joint position, motion speed and bone characteristic) generated in the data preprocessing stage. The results show that the enhanced adjacency matrix can improve each input feature, which further proves its universality and effectiveness.

#### 4.3.2. Bottleneck Block Structure and Multi-Stream Input Data

In this subsection, we describe our testing of the introduced bottleneck block structure and the effectiveness of multi-stream input data. The experimental results are shown in Table 2. We first tested the contribution of the bottleneck block structure to an EAMGCN; the results show that the bottleneck block significantly reduces the number of model parameters without a large impact on the model’s recognition accuracy. Then, the contribution of multi-stream input data to an EAMGCN was tested; the results show that the use of the multi-stream input data after data preprocessing can improve the recognition performance of the model.

#### 4.3.3. Feature Selection Fusion Module

In this subsection, we describe out testing of the effectiveness of the feature selection fusion module described in Section 3.5, and the experimental results are shown in Table 3. The experimental results demonstrate that the proposed feature selection fusion module can help improve the recognition performance of the model. 

#### 4.3.4. Combinatorial Attention Mechanism

In this subsection, we describe our testing of the effectiveness of the combinatorial attention mechanism proposed in Section 3.6, and the experimental results are shown in Table 4, where MSID and FSFM represent multi-stream input data and feature selection fusion module, respectively. ST means that the input branch used the spatial–temporal attention module alone, CA means that the input branch used the ST and FSFM modules, and the mainstream network used LAM modules simultaneously. According to the experimental results, the performance of the partial combination ST+FSFM wherein the input branch combines with the spatial–temporal attention module and the feature selection fusion module is better than that when using only one of them, which is mainly due to the fact that the spatial–temporal attention module can first extract rich feature information, and then the feature selection fusion module can obtain richer identification features. The performance of the CA combination outperforms all those adding single modules and partial combinations of modules in terms of recognition accuracies despite a small increase in the number of parameters.

### 4.4. Comparison with SOTA Methods

To verify the comprehensive performance of the CA-EAMGCN model, we compared the final model with several previous advanced methods on the NTU RGB+D 60 and 120 datasets.

#### 4.4.1. Comparison with Traditional Deep Learning Methods

As the NTU RGB+D 120 dataset is a newly released dataset, traditional deep learning methods have rarely been used on this dataset to train recognition models, so we mainly compare the proposed model with traditional deep learning methods on the NTU RGB+D 60 dataset. The comparison results are shown in Table 5. It can be seen from the comparison results that our proposed model achieves better recognition performance than traditional deep learning methods (CNN-based methods and RNN-based methods), which shows that the graph convolutional network can make better use of the spatial relationship between joints.

#### 4.4.2. Comparison with the Other Existing Methods Based on GCNs

The proposed model was compared with other existing GCN-based methods, and the comparison results are shown in Table 6. Several typical comparisons are as follows: (1) The proposed recognition model achieved good performance, that is, the recognition accuracies of the two classification benchmarks cross-subject (CS) and cross-view (CV) of the NTU RGB+D 60 dataset are 90.22% and 95.42%, respectively; the recognition accuracies of cross-subject (Csub120) and cross-setup (Cset120) classifications of the NTU RGB+D 120 dataset are 84.88% and 86.14%, respectively. (2) Compared with ST-GCN [12], the most popular skeleton-based action recognition model, the recognition accuracies of the two classification benchmarks CS and CV of the NTU RGB+ D 60 dataset are improved by 8.72% and 7.12%, respectively. (3) Compared with another popular skeleton-based action recognition model 2s-AGCN [16], our proposed recognition model has also shown significant advantages. (4) Compared with the recognition model PA-ResGCN-B19 [17], the accuracies of the proposed recognition model are slightly lower, but the number of parameters in our model for each benchmark is much lower than that of the PA-ResGCN-B19 model. (5) Compared with the latest recognition model LDT-Net [13], the recognition accuracy of the proposed model is 1.12% higher than that of the latter on the CS classification benchmark and significantly outperforms that of the latter on the CV classification benchmark of the NTU RGB+D 60 dataset. However, the number of parameters in this model is superior to our proposed method.

#### 4.4.3. Comparison with the Other Existing Methods Based on UAV-Human Dataset

We performed experiments on two benchmarks of the UAV-Human dataset and compared them with SOTA methods. The results are shown in Table 7. It can be seen that our proposed model achieves better recognition performance than other existing methods based on the UAV-Human dataset, which demonstrates the good universality of the proposed model.

In addition to the comparison results regarding accuracy, we can also see from Table 5, Table 6 and Table 7 that the number of parameters of the proposed model is also highly competitive.

### 4.5. Discussion

**Figure 5:**
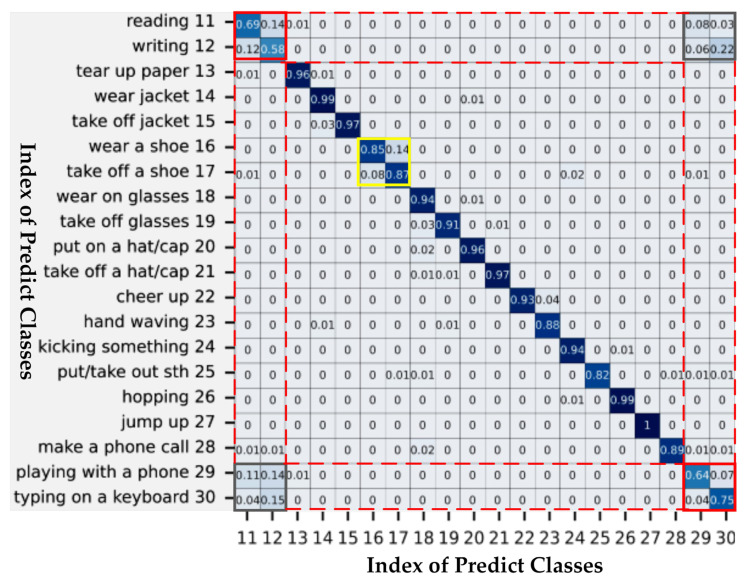
Confusion matrix of CA-EAMGCN model on the classification standard CS. The numbers on the axes represent the index of predicted classes, and the solid yellow and red boxes represent two groups of similar actions.

Although our proposed model achieved good recognition performance on two large-scale datasets, there are still some action categories that are difficult to accurately identify. As shown in Figure 5, we record the confusion matrix of partial actions on the classification benchmark CS. From Figure 5, two groups of similar movements can be observed. The first group is marked with a yellow box, including two actions of “putting on shoes” and “taking off shoes”. It can be found that these two movements are similar in spatial motion, but opposite in temporal motion. The second group, marked with two red boxes, included “reading”, “writing”, “playing on a mobile phone” and “typing on a keyboard”, all four of which are found to be performed mainly through slight movements of two hands. For the first group, the recognition accuracy can be improved by more precisely extracting the identification features at the temporal level. For the second group, the relatively low accuracy is due to the lack of typical joints that can represent the subtle changes of two hands. As a result, although the use of the adjacency matrix enhancement strategy improves the extraction of global features, our model captures very limited critical information about both hands.

**Figure 6:**
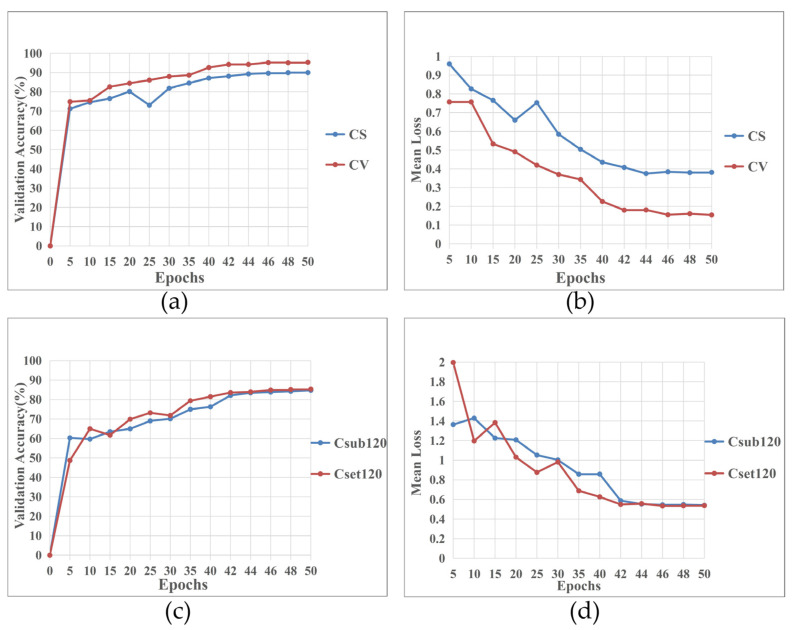
The relationships of validation accuracy and mean loss of CA-EAMGCN vs. the epochs of training on NTU RGB+D 60 and 120 datasets. (**a**) The relationship between validation accuracy and training epochs on the NTU RGB+D 60 dataset. (**b**) The relationship between mean loss and training epochs on the NTU RGB+D 60 dataset. (**c**) The relationship between validation accuracy and training epochs on the NTU RGB+D 120 dataset. (**d**) The relationship between mean loss and training epochs on the NTU RGB+D 120 dataset.

In order to show the efficiency of our proposed model in the temporal dimension, we recorded the relationship curves of the verification accuracy and mean loss of the model vs. the epochs of training, as shown in Figure 6, where subfigure (a) represents the relationship between validation accuracy and training epochs on the NTU RGB+D 60 dataset, subfigure (b) represents the relationship between mean loss and training epochs on the NTU RGB+D 60 dataset, subfigure (c) represents the relationship between validation accuracy and training epochs on the NTU RGB+D 120 dataset, and subfigure (d) represents the relationship between mean loss and training epochs on the NTU RGB+D 120 dataset. It can be observed from Figure 6 that the validation accuracy and mean loss of our proposed model gradually becomes stable when it is trained for about 42 epochs on NTU RGB+D 60 and 120 datasets. This is mainly due to the fact that fewer parameters need to be adjusted in the training process of our model, resulting in faster convergence speed and a more efficient model.

**Figure 7:**
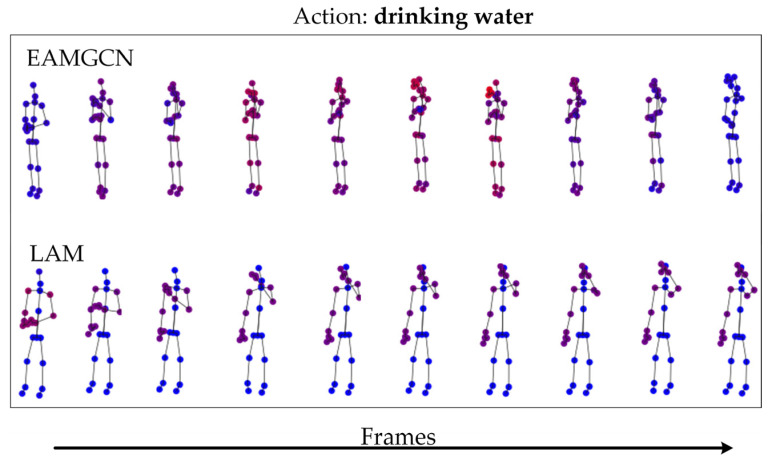
Activated joints. The red joints denote the activated joints, while the blue ones represent non-activated joints.

In order to show the workflow of our proposed model, inspired by [13], the joint activation maps of the skeleton sequence are calculated by the technique of class activation maps [41]. As shown in Figure 7, it illustrates the variance of activated joints in several sample frames. From the joint activation maps based on the enhanced adjacency matrix strategy in the top row, we find that the proposed enhanced adjacency matrix strategy can activate joints in the skeleton sequence well and improve the model’s perception ability of global information. Through the joint activation maps of LAM in the bottom row, it can be found that the LAM we proposed can find important joints in action sequences very well.

## 5. Conclusions

In this paper, we propose a novel enhanced adjacency matrix-based graph convolutional network with a combinatorial attention mechanism (CA-EAMGCN) for skeleton-based action recognition. By using an enhanced adjacency matrix, the flexibility of the graph convolutional network is increased, the model’s abilities to perceive the features of global nodes and adapt to data samples are enhanced, and the recognition performance of the model is improved. Secondly, the designed FSFM can provide an optimal fusion ratio for multiple input branches and extract more rich and discriminative features. In addition, we can focus on the important node features of human movement from multiple perspectives by using different attention mechanisms at different stages, which enhances the model’s ability to understand semantic information in different graph convolution layers. Experiments on three large datasets, NTU RGB+D 60, NTU RGB+D 120 and UAV-Human, show that our proposed model takes into account both requirements of light weight and high accuracy. However, the recognition between similar actions is still a challenging task. In our future work, we wish to extend the proposed model by placing some typical points on the human skeleton graph and using them to promote the identification of similar actions.

## Figures and Tables

**Table 1:** The effect of the enhanced adjacency matrix G*_k_* on the three input features (joint position, motion speed and bone characteristic) of the CS classification criteria, where OAM represents the original adjacency matrix and EAM represents the enhanced adjacency matrix G*_k_* introduced in Section 3.4.

Model	Methods	CS (%)		
		Joint	Velocity	Bone
ST-GCN	OAM	84.30	84.80	83.60
	EAM	86.40	85.90	85.70

**Table 2:** Verification of the bottleneck block structure and the effectiveness of multi-stream input data on the NTU RGB+D 60 dataset, where SSID means single-stream input data, IBB means introducing bottleneck block, MSID means using multi-stream input data and also introducing bottleneck block.

Model	Methods	Params (M)	Accuracy (%)	
			CS	CV
EAMGCN	SSID	3.13	86.40	93.20
	IBB	0.72	85.54	92.27
	MSID	0.80	88.60	93.42

**Table 3:** The effect of the feature selection fusion module on the NTU RGB+D 60 and 120 datasets, where MSID means multi-stream input data, FSFM means using feature selection fusion module.

Model	Methods	Params (M)	Accuracy (%)			
			CS	CV	Csub120	Cset120
EAMGCN	MSID	0.80	88.60	93.42	83.27	84.26
	FSFM	0.83	89.36	94.46	84.19	85.46

**Table 4:** Verification of the effectiveness of the combinatorial attention mechanism on the NTU RGB+D 60 dataset.

Model	Methods	Params (M)	Accuracy (%)	
			CS	CV
EAMGCN	MSID	0.80	88.60	93.42
	FSFM	0.83	89.36	94.46
	ST	0.82	89.28	94.53
	ST+FSFM	0.85	89.78	94.71
	LAM	1.06	89.59	94.57
	CA	1.10	90.22	95.42

**Table 5:** Comparison with the traditional deep learning methods (CNN-based methods, RNN-based methods) on NTU RGB+D 60 dataset.

Model	Params (M)	Accuracy (%)	
		CS	CV
HBRNN [10]	-	59.10	64.00
STA-LSTM [30]	-	73.40	81.20
TCN [9]	-	74.30	83.10
VA-LSTM [31]	24.60	79.20	87.70
Synthesized CNN [28]	-	80.03	87.21
3scale-ResNet152 [29]	-	85.00	92.30
Ind-RNN [11]	-	81.80	88.00
SRN-TSL [37]	19.07	84.80	92.40
CA-EAMGCN (ours)	1.10	90.22	95.42

**Table 6:** Comparison results with the other existing GCN-based methods on NTU RGB+D 60 and 120 datasets.

Model	Params (M)	Accuracy (%)			
		CS	CV	Csub120	Cset120
ST-GCN [12]	3.10	81.50	88.30	70.70	73.20
AS-GCN [32]	6.99	86.80	94.20	77.80	78.50
2s-AGCN [16]	6.94	88.50	95.10	82.50	84.20
AGC-LSTM [15]	22.89	89.20	95.00	-	-
PA-ResGCN-B19 [17]	3.64	90.90	96.00	87.30	88.30
3s RA-GCN [33]	6.21	87.30	93.60	81.10	82.70
Fast Convolutional [34]	1.58	89.37	94.86	85.82	87.14
2S-EGCN [20]	-	89.10	95.50	66.01	76.26
LDT-Net [13]	0.44	89.10	82.30	-	-
CA-EAMGCN (ours)	1.10	90.22	95.42	84.88	86.14

**Table 7:** Comparison results with other SOTA methods on UAV-Human dataset.

Model	Params (M)	Accuracy (%)	
		CSv1	CSv2
ST-GCN [12]	3.10	30.30	56.10
2s-AGCN [16]	6.94	34.50	66.70
EfficientGCN-B0 [18]	0.29	39.20	63.20
MKE-GCN [38]	1.46	43.80	-
STGPCN [39]	1.70	41.50	67.80
HARD-Net [40]	-	36.97	-
CA-EAMGCN (ours)	1.10	43.11	69.03

## Data Availability

The data presented in this study are available on request from the corresponding author.

## References

[1] Ju J., Zheng H., Li C., Li X., Liu H., Liu T. (2022). AGCNNs: Attention-guided convolutional neural networks for infrared head pose estimation in assisted driving system. Infrared Phys. Technol..

[2] Yang M.-H., Kriegman D., Ahuja N. (2002). Detecting faces in images: A survey. IEEE Trans. Pattern Anal. Mach. Intell..

[3] Hu W., Tan T., Wang L. (2004). A survey on visual surveillance of object motion and behaviors. IEEE Trans. Syst. Man Cybern. Part C Appl. Rev..

[4] Turaga P., Chellappa R., Subrahmanian V., Udrea O. (2008). Machine recognition of human activities: A survey. IEEE Trans. Circuits Syst. Video Technol..

[5] Ding M., Ding Y., Wu X., Wang X., Xu Y. (2021). Action recognition of individuals on an airport apron based on tracking bounding boxes of the thermal infrared target. Infrared Phys. Technol..

[6] Zhang Z. (2012). Microsoft kinect sensor and its effect. IEEE Multimed..

[7] Johansson G. (1973). Visual perception of biological motion and a model for its analysis. Percept. Psychophys..

[8] Chen S., Xu X., Yang N., Chen X., Du F., Ding S., Gao W. (2022). R-Net: A novel fully convolutional network-based infrared image segmentation method for intelligent human behavior analysis. Infrared Phys. Technol..

[9] Kim T., Reiter A. Interpretable 3d human action analysis with temporal convolutional networks. Proceedings of the IEEE Conference on Computer Vision and Pattern Recognition Workshops (CVPRW).

[10] Du Y., Wang W., Wang L. Hierarchical recurrent neural network for skeleton based action recognition. Proceedings of the IEEE Conference on Computer Vision and Pattern Recognition (CVPR).

[11] Li S., Li W., Cook C., Zhu C., Gao Y. Independently recurrent neural network (IndRNN): Building a longer and deeper RNN. Proceedings of the IEEE Conference on Computer Vision and Pattern Recognition.

[12] Yan S., Xiong Y., Lin D. Spatial temporal graph convolutional networks for skeleton-based action recognition. Proceedings of the 32nd AAAI Conference on Artificial Intelligence.

[13] Yin M., He S., Soomro T.A., Yuan H. (2023). Efficient skeleton-based action recognition via multi-stream depthwise separable convolutional neural network. Expert Syst. Appl..

[14] Kipf T., Welling M. (2016). Semi-supervised classification with graph convolutional networks. arXiv.

[15] Si C., Chen W., Wang W., Wang L., Tan T. An attention enhanced graph convolutional LSTM network for skeleton-based action recognition. Proceedings of the IEEE Conference on Computer Vision and Pattern Recognition.

[16] Shi L., Zhang Y., Cheng J., Lu H. Two-stream adaptive graph convolutional networks for skeleton-based action recognition. Proceedings of the IEEE Conference on Computer Vision and Pattern Recognition.

[17] Song Y., Zhang Z., Shan C., Wang L. Stronger, faster and more explainable: A graph convolutional baseline for skeleton-based action recognition. Proceedings of the 28th ACM International Conference on Multimedia.

[18] Song Y., Zhang Z., Shan C., Wang L. (2022). Constructing stronger and faster baselines for skeleton-based action recognition. IEEE Trans. Pattern Anal. Mach. Intell..

[19] Xie J., Miao Q., Liu R., Xin W., Tang L., Zhong S., Gao X. (2021). Attention adjacency matrix based graph convolutional networks for skeleton-based action recognition. Neurocomputing.

[20] Wang Q., Zhang K., Asghar M. (2022). Skeleton-based ST-GCN for human action recognition with extended skeleton graph and partitioning strategy. IEEE Access.

[21] Shi L., Zhang Y., Cheng J., Lu H. (2020). Skeleton-based action recognition with multi-stream adaptive graph convolutional networks. IEEE Trans. Image Process..

[22] Shahroudy A., Liu J., Ng T., Wang G. NTU RGB + D: A large scale dataset for 3D human activity analysis. Proceedings of the IEEE Conference on Computer Vision and Pattern Recognition (CVPR).

[23] Liu J., Shahroudy A., Perez M., Wang G., Duan L.-Y., Kot A. (2019). NTU RGB + D 120: A large-scale benchmark for 3D human activity understandin. IEEE Trans. Pattern Anal. Mach. Intell..

[24] Li T., Liu J., Zhang W., Ni Y., Wang W., Li Z. UAV-Human: A large benchmark for human behavior understanding with unmanned aerial vehicles. Proceedings of the IEEE/CVF Conference on Computer Vision and Pattern Recognition.

[25] Wang H., Klaser A., Schmid C., Liu C.-L. (2013). Dense trajectories and motion boundary descriptors for action recognition. Proc. Int. J. Comput. Vis..

[26] Vemulapalli R., Arrate F., Chellappa R. Human action recognition by representing 3D skeletons as points in a lie group. Proceedings of the IEEE Conference on Computer Vision and Pattern Recognition.

[27] Fernando B., Gavves E., Oramas M., Ghodrati A., Tuytelaars T. Modeling video evolution for action recognition. Proceedings of the IEEE Conference on Computer Vision and Pattern Recognition (CVPR).

[28] Liu M., Liu H., Chen C. (2017). Enhanced skeleton visualization for view invariant human action recognition. Pattern Recognit..

[29] Li B., Dai Y., Cheng X., Chen H., Lin Y., He M. Skeleton based action recognition using translation-scale invariant image mapping and multi-scale deep CNN. Proceedings of the IEEE International Conference on Multimedia & Expo Workshops (ICMEW).

[30] Song S., Lan C., Xing J., Zeng W., Liu J. An end-to-end spatio-temporal attention model for human action recognition from skeleton data. Proceedings of the AAAI Conference on Artificial Intelligence.

[31] Zhang P., Lan C., Xing J., Zeng W., Xue J., Zheng N. View adaptive recurrent neural networks for high performance human action recognition from skeleton data. Proceedings of the IEEE Conference on Computer Vision and Pattern Recognition.

[32] Li M., Chen S., Chen X., Zhang Y., Wang Y., Tian Q. Actional structural graph convolutional networks for skeleton-based action recognition. Proceedings of the IEEE Conference on Computer Vision and Pattern Recognition.

[33] Song Y., Zhang Z., Shan C., Wang L. Richly activated graph convolutional network for robust skeleton-based action recognition. Proceedings of the IEEE International Conference on Image Processing (ICIP).

[34] Nan M., Florea A.M. (2022). Fast Temporal Graph Convolutional Model for Skeleton-Based Action Recognition. Sensors.

[35] Simonyan K., Zisserman A. Two-stream convolutional networks for action recognition in videos. Proceedings of the Conference and Workshop on Neural Information Processing Systems (NIPS).

[36] Baradel F., Wolf C., Mille J. Human action recognition: Pose-based attention draws focus to hands. Proceedings of the IEEE International Conference on Computer Vision (ICCV).

[37] Si C., Jing Y., Wang W., Wang L., Tan T. Skeleton-based action recognition with spatial reasoning and temporal stack learning. Proceedings of the European Conference on Computer Vision (ECCV).

[38] Yang S., Wang X., Gao L., Song J. MKE-GCN: Multi-modal knowledge embedded graph convolutional network for skeletonbased action recognition in the wild. Proceedings of the IEEE International Conference on Multimedia and Expo (ICME).

[39] Tan Z., Zhu Y., Liu B. (2023). Learning spatial-temporal feature with graph product. Signal Process.

[40] Li T., Liu J., Zhang W., Duan L. (2020). Hard-net: Hardness-aware discrimination network for 3D early activity prediction. Proceedings of the European Conference on Computer Vision.

[41] Zhou B., Khosla A., Lapedriza A., Oliva A., Torralba A. Learning Deep Features for Discriminative Localization. Proceedings of the IEEE Conference on Computer Vision and Pattern Recognition (CVPR).

